# AurkA inhibitors enhance the effects of B-RAF and MEK inhibitors in melanoma treatment

**DOI:** 10.1186/s12967-014-0216-z

**Published:** 2014-07-31

**Authors:** Emilia Caputo, Roberta Miceli, Maria Letizia Motti, Rosarita Taté, Federica Fratangelo, Gerardo Botti, Nicola Mozzillo, Maria Vincenza Carriero, Ernesta Cavalcanti, Giuseppe Palmieri, Gennaro Ciliberto, Giuseppe Pirozzi, Paolo Antonio Ascierto

**Affiliations:** 1 Institute of Genetics and Biophysics –I.G.B., A. Buzzati-Traverso–, CNR, Via Pietro Castellino, 111, Naples, I-80131, Italy; 2 Istituto Nazionale Tumori Fondazione G. Pascale, Via M. Semmola, Naples, I-80131, Italy; 3 Dipartimento di Scienze Motorie e del Benessere, Università degli Studi di Napoli ‘Parthenope’, Via Ammiraglio Ferdinando Acton, 38, Naples, I-80133, Italy; 4 Unit of Cancer Genetics, Institute of Biomolecular Chemistry (ICB-CNR), Traversa La Crucca, 3 - Baldinca Li Punti, Sassari, I-07100, Italy

**Keywords:** Melanoma, Aurora A kinase, Targeted therapy, Combined therapy, 3D-human skin reconstruction model

## Abstract

**Background:**

Aurora kinase A (AurkA) is over-expressed in melanoma and its inhibition has been observed to limit tumor growth, suggesting a potential role in melanoma treatment.

**Methods:**

A human melanoma cell line with the B-RAF (V600E) mutation (A375mel) was exposed to B-RAF inhibitor (GSK2118436), MEK inhibitor (GSK1120212) and AurkA inhibitor (MLN8054) as single agents or in various combinations (BRAF plus AurkA inhibitor, MEK plus AurkA inhibitor or triple combination BRAF plus MEK plus AurkA inhibitor). Cell proliferation was assessed using xCELLigence technology. Total protein extracts were examined for p53 and c-Myc protein expression by Western blot analysis. Drug anti-tumor effects were further assessed using a 3D-human melanoma skin reconstruction model, in which tissues were incubated with serum-free medium containing control, B-RAF plus MEK inhibitor, MEK plus AurkA inhibitor or the triple combination.

**Results:**

AurkA inhibitor plus B-RAF inhibitor, AurkA inhibitor plus MEK inhibitor or triple combination had a markedly greater anti-proliferative effect on A375 (BRAFV600E) melanoma cells than single agents. In the 3D human skin model, the triple combination had a greater anti-tumor effect at the epidermal/dermal junction than control or either double combination. However, S-100 and Ki-67 positively stained spindle-shaped cells were detected in the dermal stratum, suggesting the presence of alive and proliferating melanoma cells.

**Conclusions:**

These findings provide new prospects for melanoma research, including combined B-RAF/AurkA inhibition for B-RAF mutated melanomas and MEK/AurkA inhibitor combination for patients without B-RAF mutations. Moreover, for the first time, we have shown that a B-RAF, MEK and AurkA inhibitor triple drug combination offers increased efficacy against melanoma cell growth and might be considered as a potential treatment strategy for enhancing clinical response in melanoma. However, although this triple drug combination was more effective at the epidermal/dermal junction, the suggested presence of alive and proliferating melanoma cells in the dermal stratum could result in drug resistance and disease recurrence. Molecular characterization of these dermal cells may be critical for the development of novel therapeutic strategies.

## Background

Melanoma is a highly aggressive skin cancer originating from melanocytes that is dramatically increasing in incidence worldwide [1],[2]. Several molecular alterations associated with melanoma have been identified and have provided new targets for therapy. In particular, gene mutations in the RAS/RAF/MEK/ERK or mitogen-activated protein kinase (MAPK) pathway have been found to be highly prevalent in melanomas. For example, around 40 different mutations in the B-RAF gene, encoding for a serine-threonine kinase, have been identified. The most frequent activating mutation (V600E-B-RAF) has been found in exon 15 and results in the substitution of valine for glutamic acid at position 600 of the BRAF protein [3],[4]. This mutation represents around 90% of BRAF mutations described in melanoma and occurs in approximately 50% of melanoma cases [5]. Melanomas carrying a B-RAF V600E mutation constitutively activate the MAPK pathway, promoting cell proliferation and preventing apoptosis [6]. Mutations in the NRAS gene have also been observed in approximately 15-20% of melanomas [7]-[9]. In addition, molecular alterations in the c-KIT and GNαQ genes have been described, primarily in mucosal and uveal melanoma subtypes, respectively [10].

B-RAF (V600E) inhibitors (e.g. vemurafenib, dabrafenib) [11] were the first targeted therapy in melanoma and have been associated with significant improvements in both progression-free survival and overall survival compared with chemotherapy in patients with B-RAF mutated metastatic melanoma [12]-[16]. However, although B-RAF inhibitor therapy produced tumor responses in the majority of patients and prolonged median survival, responses were largely partial, and clinical evidence of tumor resistance typically developed at a median of 5–7 months [17],[18]. The different resistance mechanisms following B-RAF inhibitor treatment are a consequence of the activation of alternative pathways, which can be classed as MAPK-dependent [19] or MAPK-independent [20],[21]. Acquired resistance to B-RAF inhibitors has been reported to primarily result from MAPK reactivation, driven by secondary mutations in NRAS and MEK1 genes in a subset of melanoma patients [19]. Based on these data, the combination of a B-RAF inhibitor with an inhibitor of the MAPK pathway downstream from BRAF, such as a MEK inhibitor (e.g. trametinib), has been proposed in order to overcome acquired B-RAF inhibitor resistance.

It has been observed that MEK inhibition leads to decreased cell signaling and proliferation in cancer cells [22],[23]. In particular, human melanoma cell lines with N-RAS and B-RAF mutations seem to be more sensitive to MEK inhibitor treatment than melanoma cell lines with a B-RAF-mutation only [24]. While MEK inhibitor treatment is associated with improved response rate, progression free-survival and overall survival in patients with B-RAF mutant metastatic melanoma, combined B-RAF and MEK inhibitor treatment seems to provide greater improvements in progression-free survival and overall survival compared with B-RAF inhibitor monotherapy [25]. The observation that accelerated growth of lesions harboring H-RAS mutations following exposure to B-RAF inhibitors was blocked by subsequent combined B-RAF/MEK inhibitor treatment is of particular note [26], and further supports the efficacy of MEK inhibitors and their potential role in metastatic melanoma [27],[28].

Aurora kinase A (AurkA), one of the key regulators of M phase progression, has been shown to be expressed at high levels in melanoma [29]. AurkA is a member of a serine/threonine kinase family consisting of three classes (Aurk A, B, and C) that are essential components of the mitotic pathway [30]. They ensure proper chromosome assembly, formation of the mitotic spindle, and cytokinesis. Over-expression of these kinases has been observed in several tumor types, including colon, breast, prostate, pancreas, thyroid, and head and neck, and is associated with advanced clinical stage and poor prognosis [31]-[33]. AurkA inhibition has been shown to limit tumor growth, impair mitosis and induce senescence in melanoma, suggesting a potential role in the treatment of these tumors [34].

In this study, we assessed the anti-tumor effects of an Aurk A inhibitor, B-RAF inhibitor and MEK inhibitor as single agents and in various combinations in a B-RAF (V600E) mutated human melanoma cell line and a three-dimensional (3D) human skin reconstruction model in order to provide a basis for further development of novel therapeutic strategies in the treatment of melanoma.

## Methods

### Inhibitor drugs

AurkA inhibitor (MLN8054) was purchased from Selleck Chemicals (Munich, Germany), while GSK2118436 (B-RAF inhibitor) and GSK1120212 (MEK inhibitor) were kindly provided by Glaxo SmithKline (London, UK). The B-RAF and MEK inhibitors were tested at a concentration of 30 nM in all experiments while the AurkA inhibitor was tested at a concentration of 1 μM concentration.

### Cell lines and reagents

A human melanoma cell line with the B-RAF (V600E) mutation (A375mel) was kindly provided by Dr. M. Bettinotti (NIH, Bethesda, USA). The cells were cultured in RPMI 1640 medium (Lonza, Milan, Italy), supplemented with 3 mM L-glutamine (Invitrogen-Gibco®/Life Technologies, Monza, Italy), 2% penicillin/streptomycin and 10% fetal bovine serum (FBS). All cultures were incubated at 37°C in a humidified 5% CO_2_ atmosphere.

### Cell proliferation

Cell proliferation was assessed using E-16-well plates and the xCELLigence technology (Acea Bioscience, San Diego, CA, USA, distributed by Roche Diagnostics) [35]. Briefly, cells (1 × 10^3^ A375 cells/well) were seeded in E-16-well plates in complete medium and grown for 48 hours. Inhibitor drugs were then added as single agents or in different combinations (B-RAF inhibitor [GSK2118436] plus MEK inhibitor [GSK1120212], BRAF inhibitor plus AurkA inhibitor [MLN8054], MEK inhibitor plus AurkA inhibitor [MLN8054] or triple-combination of all three drugs) and the cell growth was monitored for an additional 72 hours. Microelectrodes, placed on the bottom of plates, were used to detect impedance changes proportional to the number of adherent cells and expressed as the cell index. The impedance value of each well was automatically monitored by the xCELLigence system and expressed as a cell index value. Doubling times for each cell line were calculated from the cell growth curve during the exponential phase. The experiments were conducted in triplicate and repeated twice.

### Western blot analysis

Western blot was performed according to standard procedures. Mouse monoclonal antibody against p53 (DO-1; diluted 1:1000) (Santa Cruz Biotechnology, Inc. Dallas, TX, USA), rabbit antibody monoclonal to c-Myc (1:5000; Abcam, Cambridge, UK), and rabbit polyclonal antibody against β-actin (1:1000, Cell Signaling Technology, Danvers, MA, USA) were used. Detection was achieved by HRP-conjugated anti-mouse (Biorad, Hercules, CA, USA, 1:2,000) or HRP-conjugated anti-rabbit (Cell Signaling Technology, 1:1000) antibodies. Immune complexes were visualized by an enhanced chemiluminescence system (ECL Advance™, Amersham Pharmacia Biotech, Piscataway, NJ, USA). Actin was used as a loading control. The image analysis was performed by ImageJ software (http://rsbweb.nih.gov/ij/). Results represent the means (±SEM) of three independent experiments performed in triplicate. *P*-values were determined by using t-tests and a value ≤0.005 is reported (in figures with the symbol ***).

### 3D human skin reconstruction model

A 3D culture system of a differentiated and full-thickness skin reconstruction model of A375 melanoma cells was purchased from MatTek (Ashland, MA, USA). The 3D tissues were fed through the basolateral (bottom) surface, and incubated in duplicate with serum-free medium containing DMSO 0.2% as control, B-RAF inhibitor plus MEK inhibitor, MEK inhibitor plus AurkA inhibitor or a triple-combination of all three drugs.

The medium was replenished every other day, and the 3D tissues were collected on days 0, 5, 9 and 12 from the beginning of treatment and fixed with 4% paraformaldehyde. Tissues were paraffin-embedded, serial-sectioned, deparaffinized in xylene and rehydrated through graded decreasing concentrations of alcohol. Finally, sections were stained by hematoxylin and eosin (H&E) for cell morphology analysis. For immunohistochemistry, antigen retrieval was carried out in citrate buffer (pH6) by heating in a pressure cooker. The sections were then stained with antibodies specific for the detection of S-100 (rabbit polyclonal; Dako, Milan, Italy) and Ki-67 (Mib-1clone; Dako). Appropriate positive and negative controls were included for each antibody test. Serial sections, stained with H&E, S100 and Ki-67 were observed under a 10x/0.30 NA and 40x/0.75 NA objective lens, using a DM6000B upright microscope (Leica Microsystems, Milan, Italy). Images were captured using a high-resolution Leica DFC480 camera and digitally transferred by the Leica Application Suite 4.0 software. AdobePhotoshop CS5 software was used for image analysis. Ki-67 and S-100 positively stained cells were counted in six different fields on each slide (observed under a 40x/0.75 NA objective lens using the Leica DM6000B microscope) and their quantification was performed on digital images with the ImageJ program and AdobePhotoshop CS5. Results were expressed as percentages with respect to the controls.

## Results and discussion

### Melanoma cell exposure to B-RAF, MEK and AurkA inhibitors

**Figure 1:**
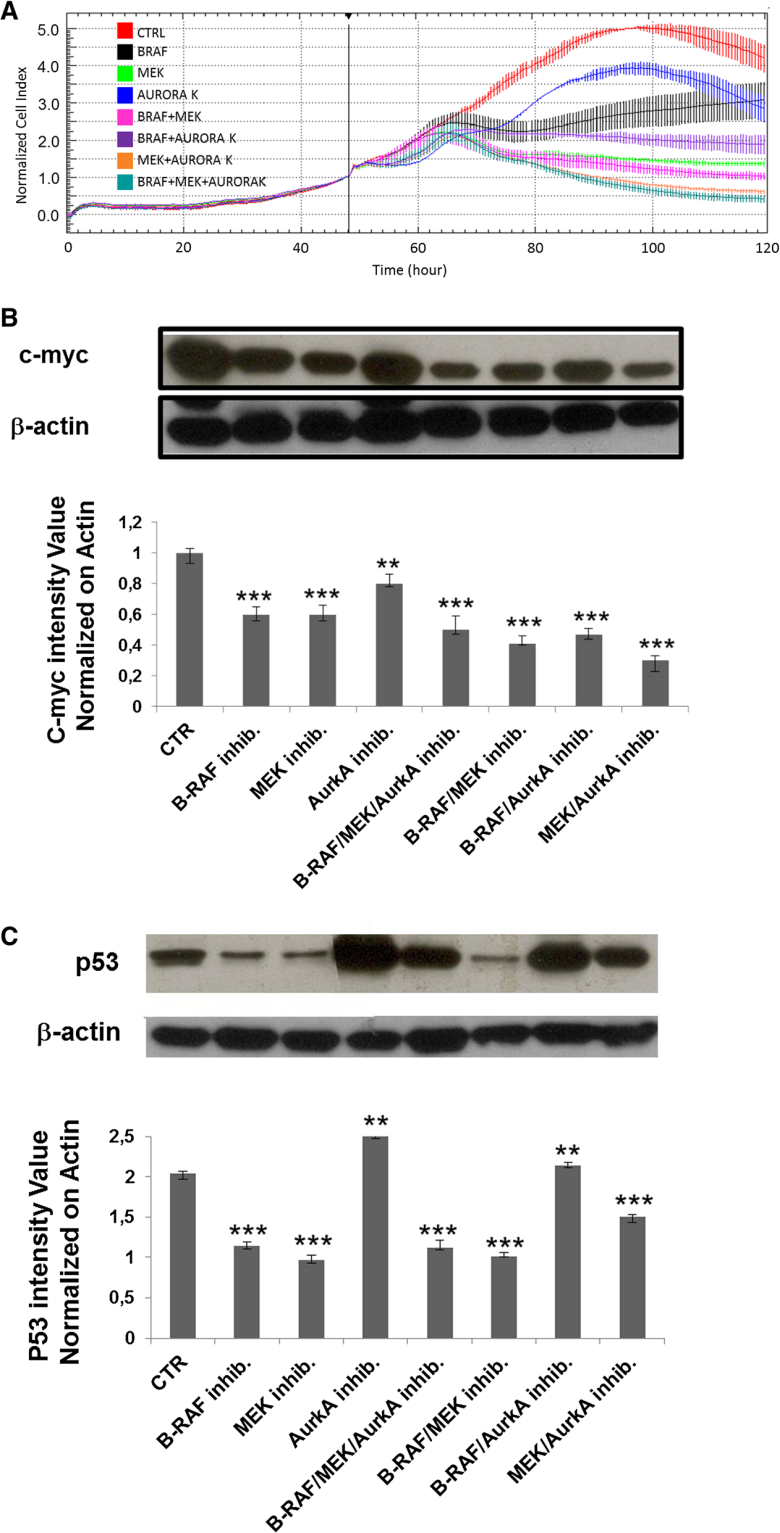
***Effect of different drugs on melanoma cell proliferation.*** Proliferation curves of A375 melanoma cells as generated by xCELLigence RTCA seeding 1×10^3^ cells/well in E-16-well plates. Cells were allowed to grow for 48 hours in complete medium before adding the indicated inhibitors, used as single agents or in different combinations, all used at 30 nM concentration except for the AurkA that was tested at 1 μM concentration. Cell growth was monitored for an additional 72 hours. Data are the mean + SD of one experiment, performed in triplicate **(A)**. Western blot analysis of c-Myc **(B)** and p53 **(C)** in melanoma cells upon different single and combined treatment at 72 hours. ***indicated P-value ≤ 0.005.

A375 (BRAFV600E) cell proliferation rates were assessed upon exposure to the different inhibitor drugs alone and in combination. As shown in Figure 1A, A375 cell proliferation rates were, as expected, reduced with B-RAF inhibitor alone and the combined B-RAF inhibitor plus MEK inhibitor. AurkA inhibitor in combination with B-RAF inhibitor and as a triple combination with B-RAF plus MEK inhibitors also reduced cell proliferation rate, although it was not very effective on cell proliferation rate, as single agent (Figure 1A). This suggests combined B-RAF/AurkA inhibition might be an alternative to B-RAF/MEK inhibition and that triple B-RAF/MEK/AurkA inhibitor therapy could be considered as a therapeutic option. Interestingly, the MEK plus AurkA inhibitor combination demonstrated higher anti-tumor efficacy than the B-RAF plus MEK inhibitor combination. This finding seems to suggest that a MEK/AurkA combination could represent an alternative treatment strategy for melanoma patients without B-RAF mutations.

To confirm that B-RAF and MEK inhibitors were inhibiting their respective proteins, we analyzed the expression of c-Myc, a downstream protein of B-RAF/MEK activated by the MAPK pathway [36]. A reduction in c-Myc protein levels in A375 melanoma cells was observed after 72 hours of exposure to B-RAF inhibitor plus MEK inhibitor, MEK inhibitor plus AurkA inhibitor and the triple drug combination (Figure 1B).

The tumor protein p53 has been reported to be phosphorylated by AurkA, leading to its increased degradation and downregulation of checkpoint-response pathways [37]. Thus, to confirm that the AurkA inhibitor (MLN8054) was inhibiting AurkA protein, we analyzed p53 protein level in the A375 cell line. After 72 hours of drug exposure, p53 protein level increased (Figure 1C). Levels of p53 were lower in cell lines exposed to double and triple drug combinations compared with single agent AurkA inhibitor, suggesting that the down-regulation of c-Myc, following B-RAF/MEK inhibition, affected p53 protein levels.

### AurkA inhibitor enhanced the effect of B-RAF and MEK inhibitors on melanoma cell growth in a 3D human skin reconstruction model

In order to further investigate the effect of AurkA inhibitors on melanoma cell growth, we used a more complex 3D human skin reconstruction model using A375 melanoma cells. Such a model offers the advantage of being more representative of the *in vivo* situation, given that cells may interact with other cells and act in a different manner when grown within a 3D matrix, while there are significant differences in cellular architecture and physiology between mouse and human skin e.g. melanocytes are mostly localized in hair follicles in mouse skin and have distinct biological properties that may differ from those of humans, in which melanocytes are mainly located at the basal layer of the epidermis.

**Figure 2:**
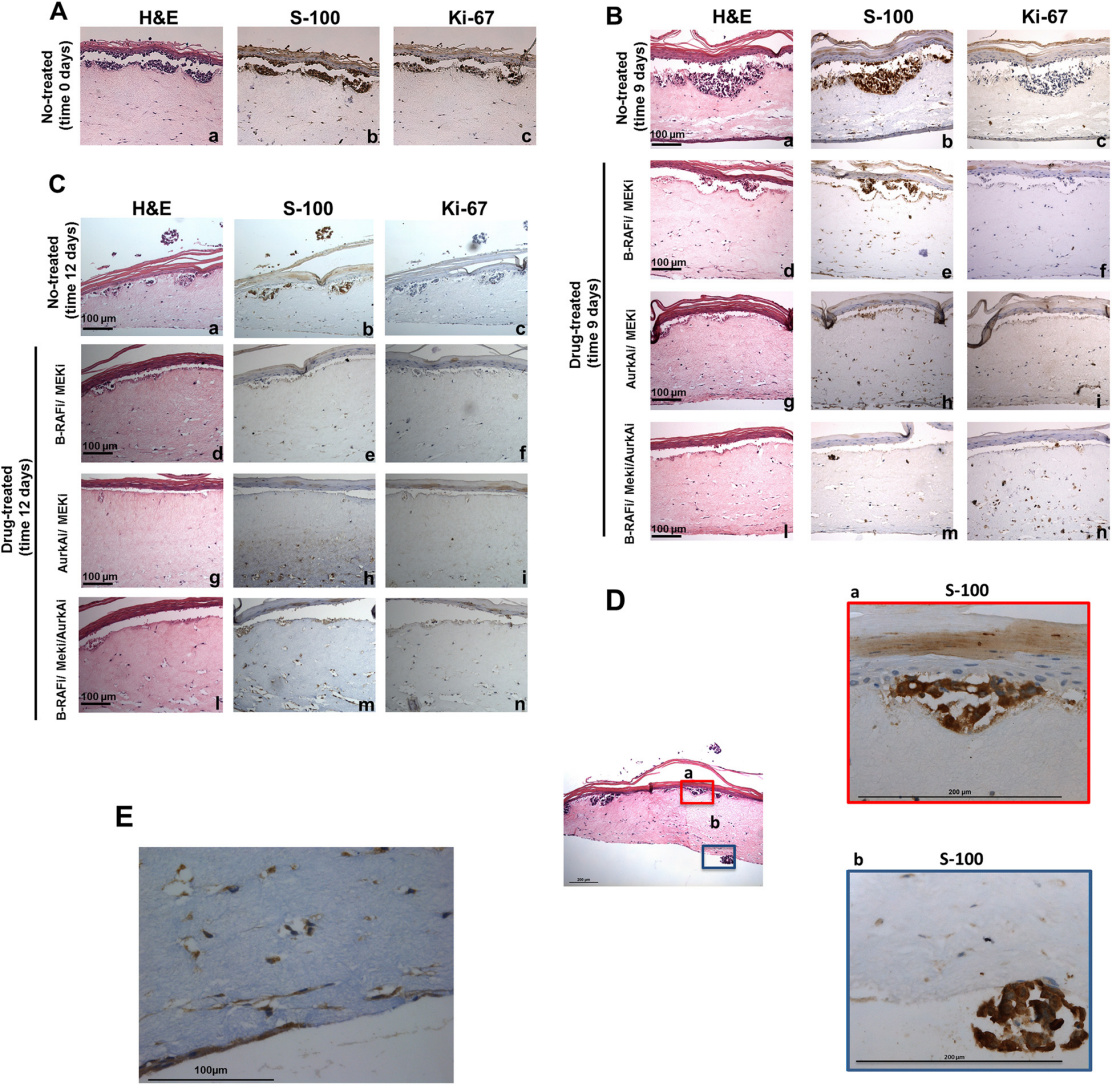
***Effect of different drugs on melanoma cell growth in human 3D tissues.*** H&E **(a)**; S-100 **(b)** and Ki-67 **(c)** staining of representative sections from 3D tissues at day 0 culture. H&E **(a)** staining showed a top bright red layer representing the epidermis; a successive layer of cells with dark blue nuclei consisting of the melanocyte layer and a bottom largely unstained layer representing the dermal stratum **(A)**; representative sections from non-treated and drug-treated 3D tissues, stained with H&E **(a, d ,g, l)**, S-100 **(b, e, h, m)** and Ki-67 **(c, f, i, n)** at day 9 **(B)** and at day 12 **(C)**; H&E, and S-100 staining of day 12 no-treated at different magnification **(D)** and a dermal stratum representative section from all 3D tissues, stained with S-100 **(E)**. The dimension bar was reported.

At baseline (day 0), H&E staining of cultures revealed keratinocytes in the upper epidermal layer, organized in the basal, spinous, granular, and corneum stratum; a second distinct layer of cells was represented by A375 melanoma cells (Figure 2A). At this early time point, this layer was only a few cells thick, with these cells distinguished by their dark nuclear staining. A third distinct layer is represented by dermal stratum consisting of fibroblast-contracted collagen. Analysis of S-100 protein expression, a marker of the melanocytic cell lineage, confirmed the presence of melanoma cells in the tissues (Figure 2A).

In the control tissue, metastatic melanocytes proliferated over time and developed nodules at the dermal/epidermal junction by day 5 (data not shown), which were further enlarged at days 9 (Figure 2B) and 12 (Figure 2C), as confirmed by their S-100 and Ki67 immunostaining. In contrast, in tissues treated with the triple drug combination there were fewer tumor nodules close to the epidermis with less invasion of the dermal structures at day 9 (Figure 2B). Exposure to B-RAF inhibitor plus MEK inhibitor or MEK inhibitor plus AurkA inhibitor provided results intermediate between control and the three drugs combined, as showed in Figure 2B; however, less tumor nodules were observed in tissues treated with the MEK plus AurkA inhibitors compared with B-RAF plus MEK inhibitors.

At day 12, the effect of the triple drug combination on melanoma growth was more pronounced (Figure 2C). Clusters of melanoma cells were detected on the other side of the dermal stratum in non-treated tissue, as shown in detail in Figure 2D, suggesting vertical growth tumor development.

Interestingly, in all treated and non-treated tissues, individual proliferating melanoma cells were observed infiltrating the dermis, as suggested by their Ki-67 (data not shown) and S-100 antigen expression (Figure 2E). These cells showed a different morphology to those observed at the dermal/epidermal junction. The small nest cells were polygonal in shape and remained confined at the junction site (Figure 2D, section a). In comparison, the individual melanoma cells in the dermal stratum appeared as spindle-shaped cells connected to one another and forming a 3D-cellular network (Figure 2E). These data suggest that melanoma dermal cells might be more able to escape the drug anti-tumor effect than small nest melanoma cells localized at the epidermal/dermal junction. The observation that the drugs penetrated to the other side of the tissues from the dermal stratum and killed the small nest cells suggests that the ability of individual melanoma dermal cells to escape the drug effect was dependent on their intrinsic properties and not on the dermal stratum being a barrier to drug exposure.

It has been previously reported that the epithelial-to-mesenchymal transition (EMT) mechanism plays an important role in promoting chemoresistance, invasion, and stem cell-like properties [38],[39]. The observed different shape of the cells suggested a potential EMT in these tissues, and it may explain the more resistant phenotype of these cells to the drugs used. This provides support to the idea that specific targeting of EMT could potentially serve to decrease metastasis and overcome drug resistance. However, further studies are needed to explain the potential role of these cells.

**Figure 3:**
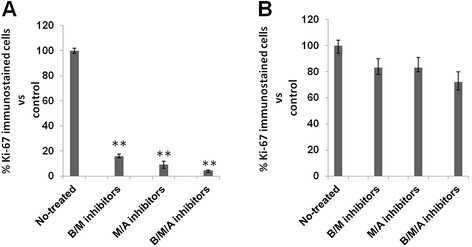
***Quantitative analysis of Ki-67 immune-histochemical results.*** Quantitative analysis of the immunohistochemical results depicting the differences in the expression of Ki-67 in 3D tissue cultures treated with different drug combination or medium without drugs as control (non-treated) at epidermal/dermal junction site **(A)** and in dermal stratum **(B)**. Results are expressed in percentage of immune-stained cells with respect to control. **p ≤ 0.01vs control.

Observations at both the epidermal/dermal junction and the dermal stratum were also quantified with the densitometric results shown in Figure 3A and B, respectively. To determine whether the decrease in S100-positive cells might account for the inhibition of melanocyte proliferation, tissue sections were stained with the proliferation marker Ki-67. This clearly revealed a decrease in the number of proliferative cells at the epidermal/dermal junction site after triple drug exposure compared with control. Intermediate results were obtained with both double drug combinations. Quantitative analysis of the Ki-67 immunohistochemical results are presented in Figure 3A. No significant differences were found in Ki-67 immunostained cells after triple or either double drug treatments at the dermal stratum compared with control (Figure 3B).

## Conclusions

Our findings provide new prospects for melanoma research, suggesting that combined B-RAF/AurkA inhibition might offer a therapeutic alternative to B-RAF/MEK inhibition for B-RAF mutated melanomas, while a MEK/AurkA inhibitor combination could represent a possible option for patients without B-RAF mutations. Moreover, for the first time, we have shown that a triple drug combination comprising of inhibitors of B-RAF, MEK and AurkA offers increased efficacy against melanoma cell growth and might be considered as a potential treatment strategy for enhancing clinical response in melanoma. However, although this triple drug combination was more effective as anti-tumor therapy at the epidermal/dermal junction, it seemed to leave individual S-100 and Ki-67 positively stained spindle-shaped melanoma cells alive and proliferating in the dermal stratum, which may result in drug resistance and disease recurrence. Molecular characterization of these dermal cells may be critical in providing additional tools for the development of novel therapeutic strategies.

## Abbreviations

MAPK: Mitogen activated protein kinases

AurkA: Aurora kinase A

## Competing interests

All authors have disclosed any financial competing interests.

PAA has/had a consultant/advisory role for Bristol Myers-Squibb, Roche-Genentech, Merck Sharp & Dohme, GSK, Ventana, and Novartis. He received research funds from Bristol Myers-Squibb, Roche-Genentech, and Ventana. He also received honoraria from Bristol Myers-Squibb, Roche-Genentech, and GSK.

## Authors’ contributions

MLM, MVC and FF carried out the cell proliferation assays. RM carried out the molecular studies by western blot analysis and helped to draft the manuscript. RT fixed the 3D tissues, which were then paraffin embedded and serial sectioned for immunohistochemical analysis, and performed the photos of the immunostained tissue samples. GB. ErC, performed 3D tissue immunostaining. PA, NM, GPal, GC and GPir participated in the design of the study. EmC conceived of the study, and participated in its design and coordination and drafted the manuscript. She also cultured and treated the 3D-tissues. All authors read and approved the final manuscript.
